# Supplementary material for: Hydrocarbon Degradation and Microbial Survival Improvement in Response to γ-Polyglutamic Acid Application
Source: Int J Environ Res Public Health. 2022 Nov 16;19(22):15066. doi: 10.3390/ijerph192215066 (PMC9690351; doi:10.3390/ijerph192215066)
Supplement: Supplementary file 1 [file ijerph-19-15066-s001.zip › ijerph-1980747-supplementary.pdf]

Table S1. Mean, Standard error and Confidence Intervals (±) expected values of dependent variables of heavy naphta, lubricated oil and grease in mixed effect of PGA treatment and time. C_{11}/C_{12}, C_{11}/C_{21}, C_{21}/C_{31}, Pr/Ph, C_{17}/Pr, C_{18}/Ph, C_{18}/H_{30}, C_{11}/H_{30}, C_{11}/C_{18} - geochemical ratios; EY – extraction yield; HML – hydrocarbon mass loss; EC – electrical conductivity; CFU – colony forming unit.

Variables	Time (days)	7	7	7	7	28	28	28	28	56	56	56	56	112	112	112	112
	Treatment	REF	PGA1	PGA1B	PGA10	REF	PGA1	PGA1B	PGA10	REF	PGA1	PGA1B	PGA10	REF	PGA1	PGA1B	PGA10
**Heavy naphta**																	
C_{11}/C_{12}	Mean	0.97	0.63	0.19	0.92	0.07	1.25	0.41	1.59	0.61	1.00	0.93	0.58	0.61	1.00	0.93	0.58
C_{11}/C_{12}	St. Error	0.45	0.56	1.03	0.47	1.72	0.40	0.70	0.35	0.57	0.45	0.46	0.59	0.57	0.45	0.46	0.59
C_{11}/C_{12}	CI(-95)	0.40	0.21	0.03	0.37	0.00	0.57	0.10	0.80	0.20	0.42	0.37	0.18	0.20	0.42	0.37	0.18
C_{11}/C_{12}	CI(+95)	2.36	1.90	1.42	2.29	1.98	2.74	1.61	3.19	1.87	2.41	2.30	1.83	1.87	2.41	2.30	1.83
C_{11}/C_{21}	Mean	43.43	10.47	2.90	33.07	0.00	0.01	0.00	0.01	0.00	0.00	0.01	0.01	0.00	0.00	0.01	0.01
C_{11}/C_{21}	St. Error	0.07	0.14	0.26	0.08	16.53	4.99	6.57	6.29	7.98	6.77	5.01	5.67	7.98	6.77	5.01	5.67
C_{11}/C_{21}	CI(-95)	38.02	7.98	1.73	28.40	0.00	0.00	0.00	0.00	0.00	0.00	0.00	0.00	0.00	0.00	0.00	0.00
C_{11}/C_{21}	CI(+95)	49.61	13.73	4.85	38.52	86.79	142.00	1812.69	1137.47	19415.95	2544.53	145.79	417.20	19415.95	2544.53	145.79	417.20
C_{21}/C_{31}	Mean	4.32	2.67	3.51	2.13	1.47	3.55	5.33	5.99	4.69	4.10	4.62	2.25	4.69	4.10	4.62	2.25
C_{21}/C_{31}	St. Error	0.22	0.27	0.24	0.31	0.37	0.24	0.19	0.18	0.21	0.22	0.21	0.30	0.21	0.22	0.21	0.30
C_{21}/C_{31}	CI(-95)	2.83	1.56	2.20	1.17	0.72	2.23	3.65	4.18	3.13	2.66	3.07	1.26	3.13	2.66	3.07	1.26
C_{21}/C_{31}	CI(+95)	6.58	4.57	5.60	3.89	3.04	5.65	7.79	8.57	7.03	6.32	6.94	4.04	7.03	6.32	6.94	4.04
EY	Mean	1.03	0.07	0.04	0.12	0.11	0.03	0.04	0.03	0.04	0.05	0.05	0.09	0.04	0.04	0.04	0.04
EY	St. Error	0.44	1.71	2.21	1.28	1.33	2.48	2.24	2.73	2.20	1.97	2.02	1.50	2.37	2.20	2.12	2.34
EY	CI(-95)	0.44	0.00	0.00	0.01	0.01	0.00	0.00	0.00	0.00	0.00	0.00	0.00	0.00	0.00	0.00	0.00
EY	CI(+95)	2.45	1.96	3.11	1.50	1.53	4.20	3.20	5.67	3.07	2.44	2.56	1.68	3.71	3.10	2.84	3.57
HML	Mean	9.01	12.19	13.14	13.21	11.30	14.34	14.67	15.92	11.49	14.53	14.14	16.61	12.66	14.57	14.44	15.86
HML	St. Error	0.15	0.13	0.12	0.12	0.13	0.12	0.12	0.11	0.13	0.12	0.12	0.11	0.13	0.12	0.12	0.11
HML	CI(-95)	6.73	9.48	10.32	10.38	8.71	11.38	11.66	12.78	8.87	11.54	11.20	13.40	9.90	11.58	11.47	12.73
HML	CI(+95)	12.06	15.66	16.73	16.81	14.67	18.08	18.44	19.84	14.88	18.28	17.85	20.60	16.20	18.33	18.19	19.76
EC	Mean	19.59	10.88	8.46	8.59	10.99	8.60	7.85	8.82	11.77	8.40	7.15	9.65	12.53	10.10	6.44	8.06
EC	St. Error	0.10	0.14	0.15	0.15	0.13	0.15	0.16	0.15	0.13	0.15	0.17	0.14	0.13	0.14	0.18	0.16
EC	CI(-95)	16.07	8.34	6.26	6.37	8.44	6.38	5.74	6.57	9.12	6.21	5.15	7.28	9.78	7.66	4.56	5.92
EC	CI(+95)	23.88	14.19	11.44	11.59	14.32	11.60	10.73	11.85	15.20	11.37	9.92	12.80	16.05	13.30	9.10	10.97
pH	Mean	5.94	6.33	6.37	6.30	6.23	6.42	6.50	6.48	6.23	6.63	6.58	6.46	6.09	6.53	6.57	6.56
pH	St. Error	0.18	0.18	0.18	0.18	0.18	0.18	0.18	0.18	0.18	0.17	0.17	0.18	0.18	0.17	0.17	0.17
pH	CI(-95)	4.15	4.47	4.50	4.44	4.38	4.54	4.61	4.59	4.38	4.72	4.68	4.58	4.27	4.64	4.66	4.66
pH	CI(+95)	8.51	8.97	9.01	8.93	8.85	9.07	9.16	9.14	8.85	9.32	9.26	9.12	8.69	9.21	9.24	9.24
**Lubrication oil**																	
Pr/Ph	Mean	0.68	0.65	0.66	0.71	0.59	0.64	0.59	0.63	0.67	0.66	0.63	0.63	0.62	0.58	0.62	0.70

Pr/Ph	St. Error	0.54	0.62	0.55	0.53	0.58	0.56	0.58	0.56	0.55	0.55	0.56	0.57	0.57	0.59	0.57	0.54
Pr/Ph	CI(-95)	0.23	0.19	0.23	0.25	0.19	0.22	0.19	0.21	0.23	0.22	0.21	0.21	0.20	0.18	0.20	0.24
Pr/Ph	CI(+95)	1.97	2.19	1.94	2.01	1.84	1.92	1.85	1.91	1.95	1.94	1.90	1.90	1.88	1.84	1.89	1.99
Pr/C_{17}	Mean	0.74	0.72	0.71	0.75	0.73	0.90	0.86	0.91	0.88	1.02	0.98	1.02	0.93	1.10	1.07	1.16
Pr/C_{17}	St. Error	0.52	0.59	0.53	0.52	0.52	0.47	0.48	0.47	0.48	0.44	0.45	0.44	0.46	0.43	0.43	0.41
Pr/C_{17}	CI(-95)	0.27	0.23	0.25	0.27	0.26	0.36	0.34	0.36	0.34	0.43	0.41	0.43	0.37	0.48	0.46	0.52
Pr/C_{17}	CI(+95)	2.05	2.29	2.01	2.06	2.04	2.27	2.22	2.28	2.24	2.42	2.38	2.43	2.31	2.54	2.50	2.62
Ph/C_{18}	Mean	0.96	0.98	0.95	0.93	0.95	1.11	1.14	1.16	1.11	1.38	1.31	1.47	1.15	1.60	1.53	1.44
Ph/C_{18}	St. Error	0.46	0.50	0.46	0.46	0.46	0.42	0.42	0.41	0.43	0.38	0.39	0.37	0.42	0.35	0.36	0.37
Ph/C_{18}	CI(-95)	0.39	0.37	0.39	0.37	0.39	0.48	0.50	0.52	0.48	0.65	0.61	0.72	0.51	0.80	0.75	0.69
Ph/C_{18}	CI(+95)	2.35	2.64	2.33	2.30	2.33	2.55	2.59	2.62	2.54	2.91	2.82	3.04	2.60	3.20	3.10	2.99
H_{30}/C_{18}	Mean	29.03	38.34	39.51	40.43	31.65	41.92	46.85	44.65	41.28	65.56	65.24	61.75	46.12	61.70	68.40	56.87
H_{30}/C_{18}	St. Error	0.08	0.08	0.07	0.07	0.08	0.07	0.07	0.07	0.07	0.06	0.06	0.06	0.07	0.06	0.05	0.06
H_{30}/C_{18}	CI(-95)	24.67	32.73	34.37	35.23	27.08	36.61	41.22	39.16	36.01	58.83	58.53	55.23	40.53	55.19	61.52	50.63
H_{30}/C_{18}	CI(+95)	34.16	44.92	45.43	46.41	36.99	47.99	53.25	50.90	47.31	73.05	72.72	69.03	52.47	68.98	76.05	63.88
C_{11}/H_{30}	Mean	1.74	1.45	1.44	1.41	0.80	0.76	0.99	1.06	0.69	0.46	0.58	0.59	0.40	0.35	0.32	0.38
C_{11}/H_{30}	St. Error	0.34	0.42	0.37	0.38	0.50	0.51	0.45	0.43	0.54	0.66	0.59	0.58	0.71	0.76	0.79	0.73
C_{11}/H_{30}	CI(-95)	0.90	0.64	0.69	0.67	0.30	0.28	0.41	0.45	0.24	0.13	0.18	0.19	0.10	0.08	0.07	0.09
C_{11}/H_{30}	CI(+95)	3.38	3.27	2.99	2.95	2.13	2.08	2.39	2.48	1.98	1.67	1.84	1.85	1.60	1.54	1.51	1.57
C_{11}/C_{18}	Mean	49.39	55.14	56.55	56.59	25.12	31.42	46.29	46.81	27.58	30.89	36.89	36.62	17.94	20.01	21.63	20.94
C_{11}/C_{18}	St. Error	0.06	0.07	0.06	0.06	0.09	0.08	0.07	0.07	0.09	0.08	0.07	0.07	0.11	0.10	0.10	0.10
C_{11}/C_{18}	CI(-95)	43.60	48.33	50.33	50.36	21.09	26.87	40.69	41.18	23.34	26.39	31.93	31.68	14.58	16.45	17.91	17.29
C_{11}/C_{18}	CI(+95)	55.96	62.92	63.54	63.58	29.92	36.74	52.65	53.21	32.59	36.17	42.61	42.33	22.06	24.35	26.11	25.37
EY	Mean	23.54	17.75	21.51	17.58	22.67	19.78	26.17	19.45	20.70	23.49	24.75	22.32	20.35	26.74	21.14	24.65
EY	St. Error	0.09	0.11	0.10	0.11	0.09	0.10	0.09	0.10	0.10	0.09	0.09	0.09	0.10	0.09	0.10	0.09
EY	CI(-95)	19.65	14.42	17.81	14.27	18.86	16.24	22.05	15.95	17.08	19.61	20.75	18.54	16.76	22.57	17.47	20.66
EY	CI(+95)	28.20	21.86	25.99	21.67	27.26	24.08	31.06	23.73	25.10	28.15	29.52	26.87	24.72	31.68	25.58	29.41
HML	Mean	0.17	0.23	1.81	0.19	0.75	2.81	2.71	3.16	1.12	4.14	3.95	4.96	1.42	2.69	2.57	3.22
HML	St. Error	1.07	0.94	0.33	1.03	0.52	0.27	0.27	0.25	0.42	0.22	0.22	0.20	0.38	0.27	0.28	0.25
HML	CI(-95)	0.02	0.04	0.94	0.03	0.27	1.67	1.59	1.93	0.49	2.69	2.55	3.35	0.68	1.58	1.49	1.97
HML	CI(+95)	1.42	1.43	3.47	1.42	2.06	4.74	4.61	5.17	2.56	6.37	6.15	7.36	2.96	4.59	4.44	5.24
EC	Mean	0.05	10.18	5.76	9.59	0.05	9.67	5.71	6.27	0.05	9.09	6.58	7.85	0.05	9.51	5.61	6.18
EC	St. Error	1.97	0.14	0.19	0.14	2.04	0.14	0.19	0.18	2.00	0.15	0.17	0.16	2.03	0.15	0.19	0.18
EC	CI(-95)	0.00	7.73	3.99	7.23	0.00	7.30	3.96	4.42	0.00	6.80	4.68	5.74	0.00	7.15	3.88	4.34
EC	CI(+95)	2.45	13.40	8.29	12.73	2.62	12.82	8.24	8.90	2.52	12.16	9.26	10.74	2.59	12.63	8.13	8.79
pH	Mean	7.66	6.63	6.68	6.57	7.20	6.74	6.69	6.66	7.34	6.70	6.63	6.65	7.43	6.61	6.77	6.74

pH	St. Error	0.16	0.17	0.17	0.17	0.17	0.17	0.17	0.17	0.17	0.17	0.17	0.17	0.16	0.17	0.17	0.17
pH	CI(-95)	5.58	4.72	4.76	4.67	5.19	4.81	4.77	4.74	5.31	4.78	4.72	4.73	5.39	4.70	4.83	4.81
pH	CI(+95)	10.51	9.32	9.38	9.25	9.98	9.45	9.39	9.36	10.14	9.40	9.32	9.34	10.25	9.30	9.48	9.44
**Grease**																	
Pr/Ph	Mean	0.39	0.41	0.40	0.39	0.42	0.45	0.45	0.41	0.38	0.40	0.42	0.39	0.38	0.43	0.38	0.43
Pr/Ph	St. Error	0.71	0.70	0.70	0.71	0.69	0.66	0.67	0.70	0.72	0.71	0.69	0.71	0.73	0.68	0.72	0.68
Pr/Ph	CI(-95)	0.10	0.11	0.10	0.10	0.11	0.12	0.12	0.10	0.09	0.10	0.11	0.10	0.09	0.12	0.09	0.11
Pr/Ph	CI(+95)	1.59	1.62	1.60	1.59	1.62	1.67	1.66	1.61	1.58	1.60	1.62	1.59	1.58	1.64	1.58	1.64
Pr/C_{17}	Mean	0.38	0.35	0.36	0.36	0.57	0.54	0.55	0.54	0.55	0.50	0.56	0.51	0.52	0.61	0.54	0.60
Pr/C_{17}	St. Error	0.73	0.75	0.74	0.75	0.59	0.61	0.60	0.61	0.60	0.63	0.59	0.63	0.62	0.57	0.61	0.58
Pr/C_{17}	CI(-95)	0.09	0.08	0.08	0.08	0.18	0.16	0.17	0.16	0.17	0.15	0.18	0.15	0.16	0.20	0.16	0.19
Pr/C_{17}	CI(+95)	1.57	1.54	1.55	1.55	1.82	1.78	1.80	1.77	1.79	1.73	1.81	1.74	1.76	1.87	1.78	1.86
Ph/C_{18}	Mean	0.60	0.58	0.58	0.58	0.82	0.75	0.75	0.78	0.81	0.74	0.80	0.76	0.79	0.80	0.79	0.84
Ph/C_{18}	St. Error	0.58	0.59	0.59	0.58	0.49	0.52	0.52	0.51	0.50	0.52	0.50	0.51	0.50	0.50	0.50	0.49
Ph/C_{18}	CI(-95)	0.19	0.18	0.18	0.19	0.31	0.27	0.27	0.29	0.31	0.27	0.30	0.28	0.29	0.30	0.29	0.32
Ph/C_{18}	CI(+95)	1.86	1.83	1.83	1.84	2.16	2.07	2.06	2.11	2.14	2.06	2.14	2.08	2.12	2.14	2.12	2.19
H_{30}/C_{18}	Mean	12.65	10.29	12.37	16.01	20.89	25.86	33.80	24.92	20.01	27.54	33.20	25.03	21.65	27.00	37.07	25.94
H_{30}/C_{18}	St. Error	0.13	0.14	0.13	0.11	0.10	0.09	0.08	0.09	0.10	0.09	0.08	0.09	0.10	0.09	0.07	0.09
H_{30}/C_{18}	CI(-95)	9.89	7.83	9.65	12.86	17.25	21.77	29.07	20.91	16.45	23.31	28.52	21.00	17.93	22.81	32.10	21.84
H_{30}/C_{18}	CI(+95)	16.19	13.52	15.88	19.93	25.31	30.72	39.30	29.70	24.34	32.55	38.66	29.82	26.13	31.97	42.81	30.81
C_{17}/C_{18}	Mean	0.63	0.67	0.64	0.63	0.60	0.63	0.61	0.60	0.56	0.59	0.60	0.59	0.57	0.57	0.56	0.61
C_{17}/C_{18}	St. Error	0.57	0.54	0.56	0.56	0.58	0.56	0.57	0.58	0.60	0.58	0.58	0.58	0.59	0.59	0.60	0.57
C_{17}/C_{18}	CI(-95)	0.21	0.23	0.21	0.21	0.19	0.21	0.20	0.19	0.17	0.19	0.19	0.19	0.18	0.18	0.17	0.20
C_{17}/C_{18}	CI(+95)	1.89	1.96	1.92	1.91	1.86	1.91	1.88	1.86	1.81	1.85	1.86	1.85	1.82	1.83	1.80	1.87
EY	Mean	17.16	13.58	13.31	14.70	18.62	15.53	21.61	15.83	16.40	20.93	21.60	20.44	11.51	23.00	21.82	21.49
EY	St. Error	0.11	0.12	0.12	0.12	0.10	0.11	0.10	0.11	0.11	0.10	0.10	0.10	0.13	0.09	0.10	0.10
EY	CI(-95)	13.89	10.71	10.47	11.69	15.20	12.43	17.90	12.70	13.20	17.28	17.89	16.83	8.89	19.16	18.09	17.79
EY	CI(+95)	21.20	17.23	16.92	18.47	22.82	19.39	26.10	19.74	20.36	25.35	26.08	24.81	14.91	27.62	26.33	25.96
HML	Mean	0.22	1.21	1.32	1.27	0.50	3.27	3.59	3.36	0.89	4.86	4.44	4.38	1.01	3.09	3.40	3.34
HML	St. Error	0.95	0.41	0.39	0.40	0.63	0.25	0.24	0.24	0.47	0.20	0.21	0.21	0.45	0.25	0.24	0.24
HML	CI(-95)	0.03	0.55	0.62	0.58	0.15	2.01	2.26	2.08	0.35	3.27	2.93	2.88	0.42	1.87	2.11	2.07
HML	CI(+95)	1.43	2.68	2.83	2.76	1.73	5.31	5.70	5.42	2.25	7.23	6.74	6.66	2.41	5.08	5.47	5.40
EC	Mean	0.15	8.24	3.81	10.26	0.10	6.82	2.60	8.63	0.11	7.35	3.25	9.04	0.13	9.69	3.15	6.74
EC	St. Error	1.16	0.16	0.23	0.14	1.42	0.17	0.28	0.15	1.35	0.16	0.25	0.15	1.25	0.14	0.25	0.17
EC	CI(-95)	0.02	6.07	2.43	7.80	0.01	4.87	1.51	6.41	0.01	5.32	2.00	6.75	0.01	7.31	1.92	4.81
EC	CI(+95)	1.44	11.18	5.96	13.49	1.60	9.54	4.48	11.63	1.54	10.16	5.28	12.10	1.48	12.84	5.16	9.44
pH	Mean	5.94	6.33	6.37	6.30	6.23	6.42	6.50	6.48	6.23	6.63	6.58	6.46	6.09	6.53	6.57	6.56

pH	St. Error	0.18	0.18	0.18	0.18	0.18	0.18	0.18	0.18	0.18	0.17	0.17	0.18	0.18	0.17	0.17	0.17
pH	CI(-95)	4.15	4.47	4.50	4.44	4.38	4.54	4.61	4.59	4.38	4.72	4.68	4.58	4.27	4.64	4.66	4.66
pH	CI(+95)	8.51	8.97	9.01	8.93	8.85	9.07	9.16	9.14	8.85	9.32	9.26	9.12	8.69	9.21	9.24	9.24

Table S2. Generalized linear mixed model (GLMM) for different parameters of heavy naphtha by treatment (PGA concentration) and time. EC – electrical conductivity; CFU – colony forming unit; C_{11}/C_{12}, C_{11}/C_{21}, C_{21}/C_{31} - geochemical ratios.

**Effect**	**df**	**Wald's Stat.**	**p**
**Extract yield**			
Intercept	1	197.96	0.0000
Treatment	3	4.50	0.21
Time	3	9.11	**0.027**
Treatment*Time	9	17.14	**0.046**
**Mass loss**			
Intercept	1	79796.78	0.0000
Treatment	3	146.17	**0.0000**
Time	3	63.05	**0.0000**
Treatment*Time	9	13.46	0.143
**pH**			
Intercept	1	1231849	0.000
Treatment	3	206	**0.000**
Time	3	75	**0.000**
Treatment*Time	9	20	**0.018**
**EC**			
Intercept	1	8901.75	0.0000
Treatment	3	104,27	**0.0000**
Time	3	17.25	**0.0006**
Treatment*Time	9	29.55	**0.0005**
**CFU**			
Intercept	1	345783	0.0000
time	4	303926205	**0.0000**
treatment	1	526201	**0.0000**
time*treatment	2	571591	**0.0000**
	df	F	p
**C_{11}/C_{12}**			
Intercept	1	129.94	0.000
Treatment	3	4.15	**0.009**
Time	3	14.84	**0.000**
Treatment*Time	9	4.27	**0.0002**
**C_{11}/C_{21}**			
Intercept	1	91.50	0.000
Treatment	3	16.27	**0.000**
Time	3	91.39	**0.000**
Treatment*Time	9	16.28	**0.000**
**C_{21}/C_{31}**			
Intercept	1	467.98.	0.000.
Treatment	3	2.21.	0.094.
Time	3	54.43.	**0.000**
Treatment*Time	9	7.16	**0.000**

Table S3. Generalized linear mixed model (GLMM) for different parameters of lubricating oil by treatment (PGA concentration) and time. EC – electrical conductivity; CFU – colony forming unit; Pr/Ph, C_{17}/Pr, C_{18}/Ph, C_{18}/H_{30}, C_{11}/H_{30}, C_{11}/C_{18} - geochemical ratios.

Effect	df	Wald's Stat.	p
**Extract yield**			
Intercept	1	25567.54	0.000
Treatment	3	4.56	0.20
Time	3	8.02	**0.045**
Treatment*Time	9	28.66	**0.0007**
**Mass loss**			
Intercept	1	3.44	0.06
Treatment	3	7.18	0.06
Time	3	12.51	**0.005**
Treatment*Time	9	6.14	0.72
**pH**			
Intercept	1	2305681	0.000
Treatment	3	1410	**0.000**
Time	3	10	**0.017**
Treatment*Time	9	109	**0.00**
**EC**			
Intercept	1	0.26	0.60
Treatment	3	79.98	**0.000**
Time	3	0.001	0.99
Treatment*Time	9	15.62	0.07
**CFU**			
Intercept	1	127223	0.000
time	1	92160	**0.000**
treatment	4	110513520	**0.000**
time*treatment	2	98612	**0.000**
**Pr/Ph**			
Intercept	1	1007.78	0.000
Treatment	3	7.65	0.053
Time	3	2.16	0.53
Treatment*Time	9	17.25	**0.044**
**C_{17}/Pr**			
Intercept	1	72.27	0.0000
Treatment	3	24.66	**0.00001**
Time	3	93.76	**0.00000**
Treatment*Time	9	9.07	0.43
**C_{18}/Ph**			
Intercept	1	76.69	0.000
Treatment	3	24.04	**0.00002**
Time	3	47.94	**0.0000**
Treatment*Time	9	13.22	0.15
**C_{18}/H_{30}**			
Intercept	1	26749.26	0.0000
Treatment	3	67.47	**0.000**
Time	3	81.99	**0.000**
Treatment*Time	9	4.36	0.88
**C_{11}/H_{30}**			
Intercept	1	59.09	0.000
Treatment	3	4.44	0.21
Time	3	184.75	**0.00**
Treatment*Time	9	9.70	0.37
**C_{11}/C_{18}**			
Intercept	1	10656.58	0.000
Treatment	3	14.18	**0.002**
Time	3	97.06	**0.000**
Treatment*Time	9	8.80	0.45

Table S4. Generalized linear mixed model (GLMM) for different parameters of grease by treatment (PGA concentration) and time. EC – electrical conductivity; CFU – colony forming unit; Pr/Ph. $C_{17/}$/Pr, $C_{18/}$/Ph, C_{18}/H_{30}, C_{11}/H_{30}, C_{11}/C_{18} - geochemical ratios.

**Effect**	**df**	**Wald's Stat.**	**p**
**Extract yield**			
Intercept	1	33518.72	0.0000
Treatment	3	46.35	**0.0000**
Time	3	20.05	**0.0001**
Treatment*Time	9	77.66	**0.0000**
**Mass loss**			
Intercept	1	104.22	0.0000
Treatment	3	73.47	**0.0000**
Time	3	67.71	**0.0000**
Treatment*Time	9	11.6	0.23
**pH**			
Intercept	1	2129357	0.0000
Treatment	3	498	**0.0000**
Time	3	18	**0.0004**
Treatment*Time	9	107	**0.0000**
**EC**			
Intercept	1	2.69	0.10
Treatment	3	160.11	**0.000**
Time	3	0.04	0.99
Treatment*Time	9	37.80	**0.00001**
**CFU**			
Intercept	1	233641	0.0000
time	4	409827492	**0.0000**
treatment	1	59733	**0.0000**
time*treatment	3	120740	**0.0000**
**Pr/Ph**			
Intercept	1	12683.13	0.0000
Treatment	3	17.84	**0.0004**
Time	3	12.92	**0.0048**
Treatment*Time	9	17.28	**0.044**
**C_{17}/Pr**			
Intercept	1	5583.70	0.0000
Treatment	3	0.49	0.91
Time	3	512.13	**0.0000**
Treatment*Time	9	18.32	**0.031**
**C_{18}/Ph**			
Intercept	1	2086.4	0.0000
Treatment	3	6.99	0.072
Time	3	455.06	**0.000**
Treatment*Time	9	10.25	0.33
**C_{18}/H_{30}**			
Intercept	1	25425.42	0.0000
Treatment	3	48.78	**0.0000**
Time	3	540.15	**0.0000**
Treatment*Time	9	71.28	**0.0000**
**C_{17}/C_{18}**			
Intercept	1	8782.38	0.0000
Treatment	3	9.7	**0.021**
Time	3	63.78	**0.0000**
Treatment*Time	9	13.59	0.13
